# Racial and ethnic inequities in occupational exposure across and between US cities

**DOI:** 10.1016/j.ssmph.2021.100959

**Published:** 2021-11-13

**Authors:** Alina S. Schnake-Mahl, Mariana Lazo, Kristina Dureja, Nahian Ehtesham, Usama Bilal

**Affiliations:** a Urban Health Collaborative, Drexel Dornsife School of Public Health, Philadelphia, PA, USA; b Department of Community Health and Prevention, Drexel Dornsife School of Public Health, Philadelphia, PA, USA; c Department of Epidemiology and Biostatistics, Drexel Dornsife School of Public Health, Philadelphia, PA, USA

**Keywords:** Occupation, Disparities, Urban health, COVID-19

## Abstract

Research suggests that racial/ethnic disparities in COVID-19 in the US are largely driven by higher rates of exposure to SARS-CoV-2 among Hispanic/Latino and Black populations. Occupational exposures play a large role in structuring risk of exposure, and essential workers are at elevated risk of COVID-19 infection. At a national-level, workers categorized as “essential” and “high-risk” are disproportionately Hispanic/Latino, but we lack analysis examining local-level racial/ethnic disparities in potential occupational exposures. Using the 2015–2019 5-year American Community Survey, we estimated disparities between the proportion of US Born Hispanic/Latino, foreign-born Hispanic/Latino, and non-Hispanic white (NHW) essential or high-risk workers in 27 of the largest metropolitan areas in the country. We found that, on average, 66.3%, 69.9%, and 62.6% of US-born Hispanics, foreign-born Hispanics, and NHW, respectively, are essential workers, while 50.7%, 49.9%, 49.5% are high exposure risk workers, respectively. The median absolute difference in proportions of US born Hispanic/Latino and NHW essential workers was 4.2%, and between foreign-born Hispanic/Latino and NHW essential workers was 7.5%, but these disparities varied widely by city. High likelihood of occupational transmission may help explain disparities in COVID-19 infection and mortality for Hispanic/Latino populations, especially foreign-born, and may also help explain heterogeneity in the magnitude of these disparities, with relevance for other acute infectious respiratory illnesses spread in the workplace.

## Highlights

• Occupational exposures play a key role in exposure risk for COVID-19.
• We find wide disparities in the proportion foreign and US born Hispanic vs non-Hispanic Whites in essential and high exposure risk occupations.
• Occupational disparities vary substantially by metropolitan area and may help explain differences in COVID-19 disparities by local area.

## 1 Introduction

COVID-19 has directly caused more than 750,000 deaths in the United States (US) by November 2021 (Dong, Du, & Gardner, 2020). Black, Indigenous, and Hispanic/Latino populations have died from COVID-19 at 3.6, 2.2, and 2.8 times that of non-Hispanic white populations, respectively (Bassett, Chen, & Krieger, 2020). While differences in mortality are a composite of differential exposure to SARS-CoV-2 and differential vulnerability to severe COVID-19, several studies have shown that inequities in mortality are mainly driven by higher rates of exposure and infection, rather than higher likelihood of death once exposed (Escobar et al., 2021; Price-Haywood, Burton, Fort, & Seoane, 2020; Rentsch et al., 2020).

Occupational exposures are a key driver of disparities in SARS-COV-2 exposure, though risk of exposure differs by occupation. In particular, essential workers are at elevated risk of exposure while working because they often cannot work from home, work in crowded and poorly ventilated conditions, and have direct exposure to the public (Dingel & Neiman, 2020; Selden & Berdahl, 2020). In the absence of strong workplace standards and enforcement, many essential workers lacked appropriate Personal Protective Equipment (PPE) in early stages of the pandemic (Hanage et al., 2020). Together, these factors combine to put essential workers at increased risk of becoming infected with SARS-CoV-2. For example, workers designated as essential in the US have a 55% higher likelihood of being COVID-19 positive than those classified as non-essential (Song, McKenna, Chen, David, & Smith-McLallen, 2021).

Essential workers are workers in occupations deemed eligible to continue working by the Department of Homeland Security during state and local “stay-at-home” and “shelter-in-place” mandates, while high-exposure risk workers are workers in occupations that regularly involve risk of exposure to infectious disease. At a national level, Hispanic/Latino, other racial/ethnic minorities, and immigrants, are disproportionately represented among essential workers (Selden & Berdahl, 2020) and among workplaces with early disease outbreaks (Bui et al., 2020); Black, Native American, and Hispanic/Latino workers are more likely than white workers to be employed in occupations with greater risk of COVID-19 exposure (Dubay, Aarons, Brown, & Kenney, 2020). Resultingly, occupational exposures may be drivers of nation-wide disparities in COVID-19 mortality (Selden & Berdahl, 2020). However, disparities in COVID-19 outcomes have differed over time, place, and populations (Schnake-Mahl & Bilal, 2021) and we do not have a clear understanding of the heterogeneity in occupational distribution of workers at elevated risk of COVID-19 infection by race/ethnicity at a local level, and particularly among large cities where COVID-19 cases were concentrated in the early months of the pandemic. In the US, much of the pandemic response was devolved down to the state and local level, and the local level is an important, but under-considered level of geography to examine in determining predictors of COVID-19 inequities (Schnake-Mahl et al., 2021).

We specifically focus on Hispanic/Latinos because of research suggesting that they have had among the highest rates of COVID-19 infection and death (Bassett et al., 2020), though risk of infection and death among this population has varied by region (Rodriguez-Diaz et al., 2020). Hispanic/Latinos been less able to work from home or stay home during shelter-in-place mandates than other racial/ethnic groups (Gould & Shierholz, 2020), and national estimates show Hispanic/Latinos are 1.3 times more likely to be essential workers than non-Hispanic White (NHW) workers (Dubay et al., 2020). We further disaggregate Hispanic/Latino populations by foreign-born versus US born, because of evidence suggesting that Hispanic/Latino immigrants experience further elevated risk of COVID-19 infection and mortality compared to the US born Hispanic/Latino population (Figueroa, Wadhera, Lee, Yeh, & Sommers, 2020; Riley et al., 2020). This elevated risk is hypothesized to be due to structural factors including concentration in high risk employment with limited job projections, barriers to accessing healthcare and vaccines (including racism and discrimination), and higher rates of housing crowding (Riley et al., 2020).

We employ racial capitalism theory to help explain racial/ethnic occupational disparities, and how structural factors pattern differential occupational exposures to infectious disease (McClure, Vasudevan, Bailey, Patel, & Robinson, 2020). Racial capitalism posits that capitalism operates within a racist system, and that therefore racism is fundamental for reproduction of the capitalist system (Robinson, 2020); this framing orients us to consider the specific and intersecting impacts of both capitalism and racism in producing occupational inequities. We extend this to consider how occupational inequities further structure inequities in workplace hazards, resulting in the overrepresentation of minoritized population in occupations at risk of exposure to the disease that causes COVID-19. Exposure risks however are not limited to COVID-19, as rates of influenza show similar patterns of racial and socioeconomic inequities (Quinn et al., 2011). Given similar transmission dynamics, occupations that put employees at high risk of SARS-CoV-2 exposure also put workers at high risk for influenza and other infectious respiratory virus exposure. As COVID-19 shifts to an endemic disease (Phillips, 2021), and considering the continuing role of influenza and other respiratory viruses in causing severe disease and mortality (Jain et al., 2015; Rolfes et al., 2018) developing interventions aimed at reducing occupational exposure and subsequent disparities in outcomes will be needed.

The objective of this paper is to conduct a descriptive analysis that categorizes occupational codes by high-exposure risk and essential worker category, and then examines racial/ethnic distribution of workers in these occupational groups, across major US metropolitan statistical areas (MSAs). We hypothesized that across all MSAs, foreign-born Hispanic/Latino workers would be most likely to work in essential and high-exposure jobs, followed by US born Hispanic/Latino and NHW workers.

## 2 Materials and methods

### 2.1 Study setting and data sources

The unit of analysis of this study were the 27 metropolitan statistical areas (MSA) in which the Big Cities Health Coalition (BCHC) member health departments are located (National Association of County and City Health Officials (NACCHO), 2021). The BCHC is an organization comprised of the largest urban health departments in the US. We operationalize the “local” environment using Core-Based Statistical Areas (specifically Metropolitan Statistical Areas, or MSAs), as geographically defined labor markets based on core population agglomerations. While commuting zones are another geographic measure of labor markets, these were developed to focus on connectivity of rural places, so are less appropriate geographic units given our focus on core economic areas (Fowler & Jensen, 2020). For brevity, we refer to MSAs by the name of the principal city in each MSA, and use the February 2013 MSA definitions (Census, 2020).

We obtained microdata from the Census Bureau's 2015–2019 five-year American Community Survey (ACS) public use microdata sample file, via IPUMS USA (Ruggles et al., 2020), and limit our study sample to the employed population, or individuals 16 or older who are part of the labor force, who identified as Hispanic/Latino or NHW. We further categorized Hispanic/Latinos as US born or foreign-born, regardless of citizenship or immigration status, because of evidence suggesting that Hispanic/Latino immigrants experience further elevated risk of infection and mortality compared to the US born Hispanic/Latino population (Figueroa et al., 2020; Riley et al., 2020) (See Appendix Fig. 1). Our final sample therefor includes observations from employed Hispanic (US and foreign born) and NHW respondents aged 16 or older in the 27 BCHC MSAs. All observations meeting the above criteria are included in our final sample. For all variables in our analysis, IPUMS carries out hot-deck missing data allocation, so data is available for all variables and observations employed in our analysis (IPUMS USA, 2021).

### 2.2 Outcome

We created two categorizations of workers: “essential workers” and “high-risk (of exposure) workers”. First, to create the “essential worker” category we applied the industry guidelines issued by the Department of Homeland Security Cybersecurity and Infrastructure Security Agency (CISA) to identify, “essential critical infrastructure workers” (Wales, 2020). We identified essential worker occupations in the ACS data using the list of Standard Occupational Classification (OCCSOC) titles and codes, linked to the CISA advisory list of Standard Occupational Classification (SOC) codes, produced by the Labor Market Information Institute (Labor Market Investigation Institute (LMI), 2020), with updates to reflect ACS non-numeric OCCSOC coding. We also examined essential worker industries, rather than occupation, as a sensitivity analysis, using the mapping created by the Centers for Disease Control and Prevention (CDC) to link North American Industry Classification System (NAICS) industry codes to essential industry designation categories (Centers for Disease Control, 2021) and then linked the NAICS codes to 2018 and onward ACS Census Industry codes (CIC) (IPUMS USA, 2018), with updates to align with the 2019 ACS Industry codes (IND). The coding defines all workers in essential industries to be essential workers, regardless of their occupation within the industry (Kerwin, Nicholson, Alulema, & Warren, 2020), and we only include industries classified as essential in our “essential” definition, not those industries classified as “mixed-essential” (e.g. industries containing essential and non-essential occupations). We created a second categorization to consider high-risk workers. We linked the ACS primary occupation and industry categories to a Bureau of Labor Statistics (BLS) O*NET survey measure that reported how frequently workers in each occupation are exposed to infection or disease at work. We replicated the methods employed by Baker et al. (see for further information on the O*NET survey) (Baker, Peckham, & Seixas, 2020), to identify occupations as “at risk” for exposure to infectious disease more than once a month. We defined “at risk” as occupations that received a score of 50–100 based on responses asking about the frequency of job exposure to disease or infection. We then merged the Standard Occupational Classification code (SOC) “at risk” codes with the ACS occupational codes to estimate the total number of individuals employed in the occupations with exposure to disease/infection more than once a month. We categorized people as “high exposure risk” if anyone worked in an “at risk” occupation. The appendix contains the categorized list of SOCs that are included in the high exposure risk category. The two categories are not mutually exclusive: workers could be categorized in both the essential and high-risk groups.

### 2.3 Statistical analysis

We computed the proportion of US born and foreign-born Hispanic and NHW workers in “high exposure risk”, “essential worker” occupations, and the proportion of “essential workers” in both “high exposure” and “low exposure” risk occupations, by MSA. All analyses included person weights to ensure calculations were representative of the noninstitutionalized population at the MSA and national levels. We created absolute disparity measures by calculating the difference in the proportion of workers in an essential or high-exposure risk occupation that are foreign-born Hispanic and US born Hispanic workers, compared to NHW workers, and estimated 95% confidence intervals of this difference. We perform Kruskal-Wallis tests to estimate whether differences in proportions by MSA are statistically significant. All analyses were conducted using R version 4.0.2.

## 3 Results

We included 2,323,346 observations across 27 MSAs, representing a total of 50,028,026 individuals (Table 1). Of the total population of any race/ethnicity, the proportion of US born Hispanic/Latino individuals ranged from 1.8% to 42.7% across MSAs (median 8.9%, Interquartile range (IQR) 3.3, 13.2%), the proportion of foreign-born Hispanics/Latinos ranged from 1.8 to 35.6% (median 10.7%, IQR 4.2, 13.3%), and proportion of NHW ranged from 29.6 to 79.1% (median 57.1%, IQR 45.1, 71.1%) (Appendix Table 2). Across MSAs, a median of 66.1% of workers were categorized as workers in essential occupations and 49.9% as high-exposure risk workers. Overall, 30.4% of workers were categorized as low exposure risk essential workers, 32.9% as high exposure risk essential workers, 17.3% as high exposure risk non-essential and 19.3% as neither high risk nor essential. The proportion of US born Hispanics/Latinos essential workers ranged from 60.5% in Washington, DC to 72% in Cleveland, with a median of 66.3% (IQR: 64.1, 67.6%); for high exposure risk, US born Hispanic/Latino proportions ranged from 46.3% (Kansas City) to 56.8% (Las Vegas), with a median of 50.7% (IQR: 49.0, 52.4%). For foreign-born Hispanics/Latinos the proportion of essential workers ranged from 62.4% in Miami to 75.1% in Minneapolis, with a median of 69.9% (IQR: 68.3, 70.7%), and for high exposure risk workers, the proportion ranged from 38.7% in Indianapolis to 59.4% in Las Vegas (median: 49.9%, IQR:46.8, 54.0%). For NHW workers, the proportion of essential workers ranged from 56.7% in Miami to 66.1% in Columbus, OH (median: 62.6%, IQR: 60.7, 63.5%), and 48.0% (Minneapolis) to 56.1% (Las Vegas) for high exposure risk workers (median 49.5%, IQR:48.9, 51.0%). We found significant differences between ethnic/nativity groups across MSAs in the proportion of workers employed in essential occupations (p=<0.001), but no significant differences for high-risk occupations across MSAs (p = 0.54).

**Table 1:** Proportion of Essential and High Exposure Risk Workers by Hispanic/Latino Ethnicity and Nativity. U.S. non-institutionalized employed population living in 27 large MSAs* (2015–2019 American Community Survey).

	US-Born Hispanic median [IQR]	Foreign-Born Hispanic median [IQR]	Non-Hispanic White median [IQR]	Total median [IQR]
Weighted population Total (N)	(14,655,561) 12.9% [4.2%, 20.5%]	(16,704,709) 13.1% [5.5%, 19.1%]	(69,750,544) 74.4% [61.4%, 89.4%]	101,110,814 100%
High Exposure Risk workers	50.6% [47.2%, 54.2%]	51.2% [47.2%, 54.2%]	49.7% [49.1%, 51.0%]	50.4% [48.7%, 52.0%]
Essential Occupations	65.2% [63.5%, 67.4%]	69.4% [68.1%, 71.0%]	62.5% [60.8%, 63.4%]	65.4% [62.7%, 68.5%]
Essential Occupations- High Risk	43.9% [42.1%, 46.3%]	40.3% [39.3%, 41.5%]	39.2% [38.4%, 40.31%]	40.4% [39.1%, 42.4%]
Essential Occupation-Low Risk	40.1% [37.9%, 42.6%]	42.2% [38.4%, 44.7%]	38.3% [37.0%, 40.1%]	40.2% [37.9%, 42.5%]

Footnote: numbers are median [Interquartile range: Quartile 1, Quartile 3]. Categorization of essential and high risk are not mutually exclusive *part of BCHC.

**Fig. 1:**
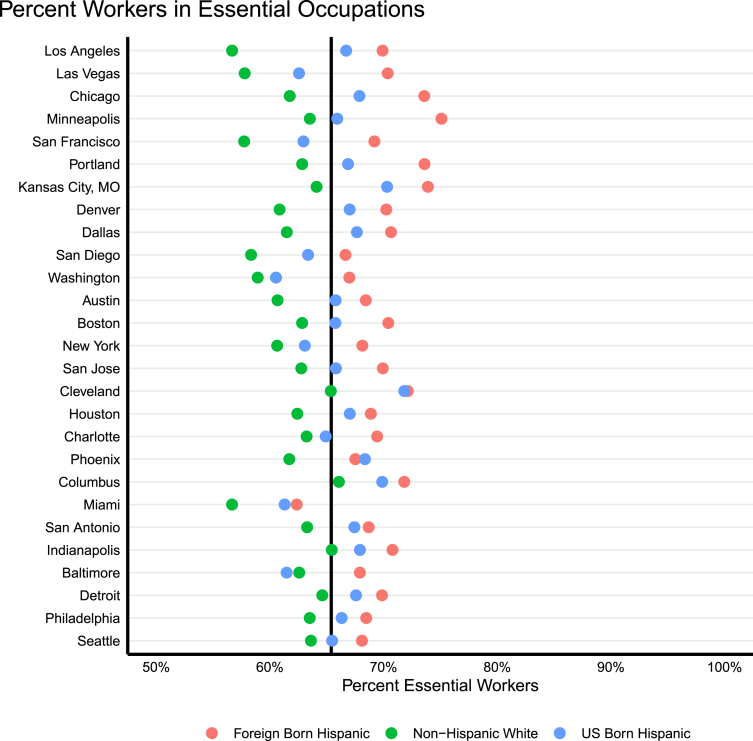
Proportion of Workers in Essential Occupations by MSA, Ethnicity and Nativity Footnote: cities are sorted by foreign-born Hispanic/Latino vs NHW absolute disparity.

We found substantial heterogeneity in the size of disparities comparing the proportion of US and foreign-born Hispanic/Latino essential or high exposure risk workers to NHW workers, though disparities ranged more for essential workers than high exposure risk, and differences were only significant across MSAs for essential workers (p<0.001). Fig. 1 shows the MSA-specific proportion of workers in essential occupations that are US born Hispanic/Latino, foreign-born Hispanic/Latino, or NHW. In all but one MSA (Baltimore, MD) the proportion of US born Hispanic/Latino essential workers was higher than the proportion of NHW essential workers, and in all MSAs the proportion of foreign-born Hispanic/Latino essential workers were higher than the proportion of NHW essential workers. The median absolute difference in proportion of US born versus NHW essential workers was 4.2.% (IQR: 2.6, 5.7%) with absolute differences ranging from a low of −1.1% (Baltimore), indicating higher proportion of NHW as essential workers, as compared to US-born Hispanic/Latinos, to as large as 10.1% in Los Angeles, indicating a higher proportion of US-born Hispanic/Latino essential workers, as compared to NHW. For foreign-born Hispanic/Latinos versus NWH, the median difference in proportion of essential workers was 7.5% (IQR: 5.7, 9.6%), ranging from a low of 4.5% (Seattle) to a high of 13.2% (Los Angeles).

**Fig. 2:**
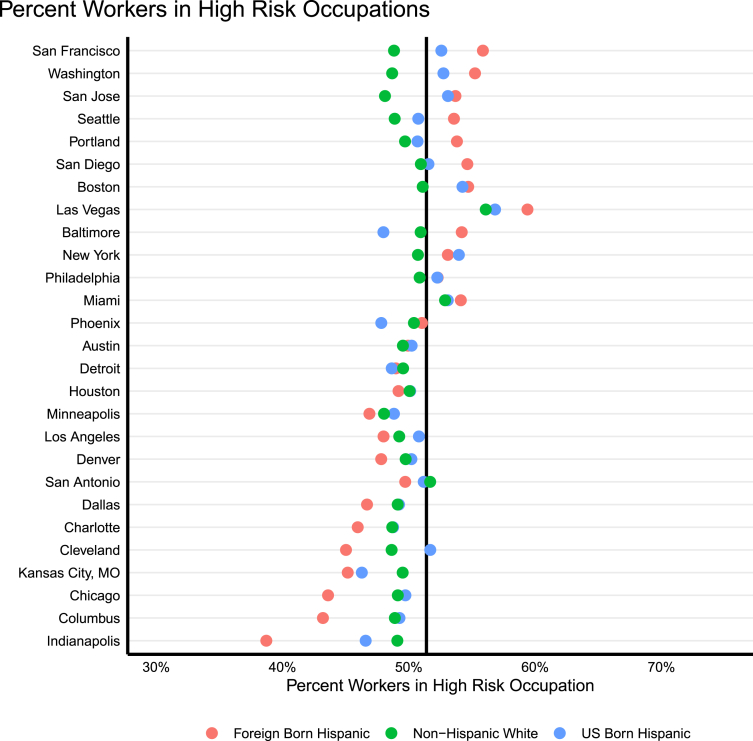
Proportion High Exposure Risk Workers by MSA, Ethnicity and Nativity Footnote: cities are sorted by foreign-born Hispanic/Latino vs NHW absolute disparity.

For high exposure risk occupations (Fig. 2), the pattern was less consistent; foreign-born Hispanics/Latinos made up the highest proportion of high exposure risk workers in twelve MSAs, US born Hispanics/Latinos in eleven, and NHW in four. For high exposure risk workers, the median absolute difference comparing US born to NHW was 0.6% (IQR: 0.05, 1.7%), with differences ranging from −3.2% (Kansas City) to 5.0% (San Jose); for foreign-born Hispanic/Latino versus NHW the median absolute difference was −0.4% (IQR: −2.2, 3.4%) with differences ranging from −10.4% (Indianapolis) to 7% (San Francisco).

A median of 81.2% of workers were employed in essential industries, with 81.1% of US born Hispanics, 86.9% of foreign-born Hispanics, and 78.2% of NHWs employed in essential industries (Appendix Table 2). Appendix Fig. 2 shows the MSA-specific proportion of workers in essential industries that are US born Hispanic/Latino, foreign-born Hispanic/Latino, or NHW. Results were similar to the analysis for essential occupations, but percentages of essential industry workers were about 20 percentage points higher, likely reflecting the broader industry categorization. The median absolute difference in proportion of US born versus NHW workers in essential industries was 3.2% (IQR: 1.8, 5.1%), and was 8.2% (IQR: 6.5, 10.4%) for foreign born versus NHW workers.

## 4 Discussion

We found that, across large metropolitan areas of the US, a large proportion of workers were categorized as workers in essential occupations, and a smaller proportion were categorized as at high-risk of exposure to infectious diseases. We found disparities in the proportion of foreign-born Hispanic/Latino and US born Hispanic/Latino essential workers versus NHW essential workers, though disparities varied substantially by MSA, and were larger for foreign-born Hispanics, while the percentage of workers at high risk of exposure did not differ on average. This finding is consistent with a national level analysis that found similar rates of high-risk exposure occupations among Hispanic/Latino and NHW workers (Hawkins, 2020).

Though the data is limited, research suggests that workplace exposure accounts for a substantial proportion of community spread of SARS-CoV-2 (Selden & Berdahl, 2020). For example, meat and poultry processing facilities have been sites of some of the largest super spreader events (Dyal, 2020). A study in the Boston area found that in the early months of the pandemic, grocery store employees with customer facing jobs were nearly five times more likely to have been infected with COVID-19 than employees in other roles (Lan, Suharlim, Kales, & Yang, 2021). Another study found increased COVID-19 hospitalization risk among construction workers who resumed work during shelter-in-place orders, compared to other occupational categories (Pasco, Fox, Johnston, Pignone, & Meyers, 2020). A Massachusetts study found that people occupied in healthcare support, transportation and material moving, and food preparation and serving had the highest age-adjusted mortality rates of any occupation, with higher death rates among Hispanic/Latino and non-Hispanic Black workers in those high risk occupations (Hawkins, Davis, & Kriebel, 2021). And, a study found that in California, excess mortality in COVID-19 was highest among Latino and Black essential workers (Chen et al., 2021).

Generally, metropolitan areas include large proportions of health care workers, occupations categorized as essential by DHS (Wales, 2020), likely helping to explain why a larger percentage of workers in the cities were categorized as essential than at the national level. The differential likelihood of exposure among US and foreign-born Hispanic essential workers, and differences in these disparities by MSA, reflects higher rates of employment among foreign-born Hispanic populations in occupations deemed essential including service, transportation, and maintenance occupations (Bureau of Labor Statistics (BLS) (2020). These national patterns are the product of structural factors (e.g. educational disparities, financial precarity, limited workplace protections and limited bargaining power, residential segregation, and discriminatory policies and practices) that create occupational segregation and limit employment opportunities for foreign born populations (Ayón, 2015; Gany, Novo, Dobslaw, & Leng, 2014). Subnational variation in occupational segregation by ethnicity and nativity likely reflect local variation in these structural factors, as well as differences in local labor markets, proportions of various racial/ethnic groups (Alonso-Villar, Gradin, & Del Río, 2013), and policies that enable or restrict access to various occupations. Our finding of substantial variation in essential and high exposure risk differences by ethnicity and nativity highlights the importance of local-level occupational differences, which may change risk factors for exposure to infectious disease, in examining and addressing health disparities.

Disparities in COVID-19 outcomes differ by geography and wave (Schnake-Mahl & Bilal, 2021) but cumulative rates of infection are consistently higher among Hispanic/Latino compared to NHW populations (Bassett et al., 2020) with the highest rates among foreign-born Hispanics/Latinos (Figueroa et al., 2020; Riley et al., 2020). Research suggests these inequities are due to higher rates of exposure and infection among Hispanic/Latino populations (Escobar et al., 2021; Price-Haywood et al., 2020; Rentsch et al., 2020). Our findings highlight one potential explanation for differences in disparities by place: much higher rates of Hispanic/Latino workers employed in essential occupations in some places, and more limited disparities elsewhere.

Our analysis has some limitations. The analysis reflects data from the 2015–2019 ACS, the most recently available data. The analysis does not reflect any changes in employment that may have occurred after 2019, including any potential impacts of the pandemic on employment. Further research should examine which specific occupations or industries drive the observed disparities, and other potential explanations for metro-specific differences in disparities. Additionally, few analyses in the US consider occupational class, instead opting to analyze income or educational differences as measure of socioeconomic status, but occupational class analysis may produce differential estimates of impact and disparities, and so deserve further exploration (Muntaner, 2019). The lack of a disparity in high-risk workers may be due to differences in occupational patterns by race/ethnicity and nativity, and researchers using this category of exposure risk may fail to identify occupational disparities in exposure risk. Importantly, our definition of workers at high-risk of exposure, based on the work by Baker et al. (Baker et al., 2020), uses self-reported perception of exposure to pathogens on the O*NET survey, collected from 2001 to 2011. The O*Net survey does not update survey responses as occupational categories change, suggesting responses may not reflect recent changes to occupational characteristics and environments. Furthermore, given new developments in knowledge about COVID-19 transmission, especially in crowded and poorly ventilated spaces, these perceptions may not entirely correlate with actual risk for COVID-19. As a key example, food preparation and serving related occupations, which have had some of the highest rates of COVID-19 cases and mortality (Dubay et al., 2020), are not classified as high exposure risk workers. Guidelines on risk of exposure should be updated to reflect the latest scientific knowledge about the transmission of COVID-19 and other airborne pathogens. Furthermore, this definition may also mask important disparities in telework and access to PPE, other factors related to occupational risk.

Our work has implications beyond the immediate pandemic. The occupational and household risk we describe are also relevant for other infectious disease such as influenza. Previous analysis showed similar patterns of disproportionate occupational exposure during the 2009 H1N1 pandemic for Hispanics/Latinos versus NHWs (Kumar, Quinn, Kim, Daniel, & Freimuth, 2012). In preparing for subsequent pandemics and annual endemic infectious disease, policies and systems should be developed to protect workers, with recognition of the racial and ethnic disparities in occupational distribution. During the COVID-19 pandemic essential workers have performed critical societal functions that have allowed non-essential workers to avoid unnecessary exposures, but the burden of this responsibility has fallen disproportionately on Hispanic/Latino households and other racial/ethnic minorities. Future research should consider replicating this analysis for 1) other minoritized groups (Black, Indigenous, Native Hawaiian and Pacific Islander, and Asians), to understand occupational disparities for additional groups that have disproportionately experienced the adverse health and economic impacts of COVID-19; and 2) other geographies and geographic delineations, including rural areas, commuting zones, and disaggregated urban and suburban (within MSA) areas, as racial/ethnic occupational patterns may differ by geographic delineation and area.

As racial capitalism highlights, solutions to occupational disparities need to address the intersecting exploitation of capitalism and racism in structuring occupational inequities (Robinson, 2020). This suggests policy solutions should not be race-neutral, but rather should target programs and policies to Hispanic/Latino individuals in areas with occupational disparities. Despite higher exposure risk, elevated mortality and disproportionate representation of minoritized populations, some essential workers were not prioritized for vaccination in many states (Schmidt et al., 2021). States, cities, and employers should ensure easy access to vaccination for employees, especially those at high risk of exposure, including by providing paid time off to receive vaccines. Worker protections, including Paid Sick Leave laws, which vary substantially in availability by city and state (Peters, Marotta, & Bramhall, 2020), can also help prevent spread of infectious disease (Pichler, Wen, & Ziebarth, 2020). For example, as COVID-19 continues and becomes endemic (Lavine, Bjornstad, & Antia, 2021) states and cities can target annual vaccination outreach programs, and consider providing hazard pay, to workers in occupations that are at elevated risk of exposure and spread. To reduce disparities, policy solutions should address the root causes of disproportionate representation of Hispanic, and particularly foreign-born Hispanics, among high exposure risk and essential occupations, such as policies that prevent discrimination and enforce existing discrimination laws, support for broad worker protections and unionization efforts, employment training opportunities, and increased social insurance.

## 5 Conclusions

This research highlights disparities in the proportion of US and foreign-born Hispanic/Latino essential workers, compared to NHW essential workers, in large MSAs across the US. We identify one disparity that may contribute to understanding observed racial and ethnic disparities in COVID-19, and potentially other respiratory diseases. Local area specific estimates of racial/ethnic disparities in occupational risk can help cities plan and target interventions to mitigate risk among specific occupational and racial/ethnic groups at elevated risk of exposure to infectious respiratory disease, and potentially help explain differences in disparities by metropolitan area.

## Declarations of interest

Alina Schnake-Mahl was holds equity, and currently consults for Cityblock Health.

## Author contributions

AS and UB conceived the study and drafted the first version of the manuscript. AS conducted the statistical analysis with support from KD, MA, and UB. NE supported the literature review. All authors reviewed the manuscript for critical intellectual content and have approved the final version.

## Funding

This study was supported by the Office of the Director of the 10.13039/100000002National Institutes of Health under award number DP5OD26429; and the 10.13039/100000867Robert Wood Johnson Foundation (10.13039/100000867RWJF) under award numbers 77644 and 78325. ML was supported by NIH/10.13039/100006545National Institute on Minority Health and Health Disparities R21MD012352-02S1. The funding sources had no role in the analysis, writing or decision to submit the manuscript.

## Declaration of competing interest

The authors declare no conflict of interest.
