# Supplementary material for: Racial and ethnic inequities in occupational exposure across and between US cities
Source: SSM Popul Health. 2021 Nov 13;16:100959. doi: 10.1016/j.ssmph.2021.100959 (PMC8590507; doi:10.1016/j.ssmph.2021.100959)
Supplement: Multimedia component 2 [file mmc2.docx]

**Appendix**

**Appendix Figure 1: Population Exclusion Flowchart**

**
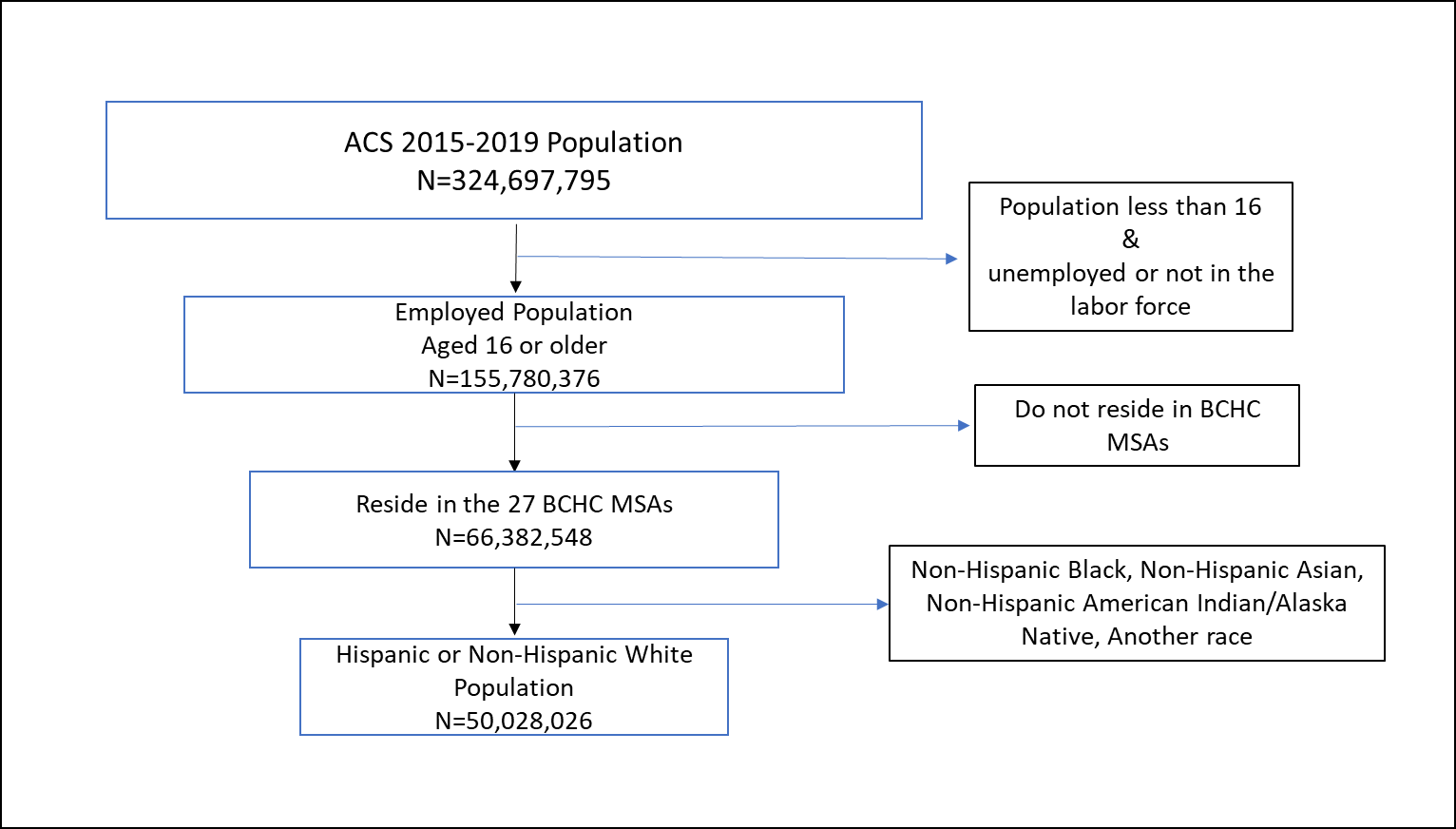
**

**Appendix Table 2: Proportion US born Hispanic, Foreign born Hispanic and Non-Hispanic White (of total population) by MSA**

| **MSA** | **Proportion US-Born Hispanic** | **Proportion Foreign-Born Hispanic** | **Proportion Non-Hispanic White** | **Proportion Other Race/**  **Ethnicity** |
| --- | --- | --- | --- | --- |
| Austin-Round Rock, TX | 18.9% | 18.9% | 54.8% | 14.8% |
| Baltimore-Columbia-Towson, MD | 2.3% | 2.3% | 59.3% | 35.2% |
| Boston-Cambridge-Newton, MA-NH | 3.5% | 3.5% | 72.6% | 17.3% |
| Charlotte-Concord-Gastonia, NC-SC | 2.8% | 2.8% | 62.1% | 28.7% |
| Chicago-Naperville-Elgin, IL-IN-WI | 10.2% | 10.2% | 56.7% | 22.3% |
| Cleveland-Elyria, OH | 3.0% | 3.0% | 74.3% | 20.7% |
| Columbus, OH | 1.8% | 1.8% | 74.9% | 21.3% |
| Dallas-Fort Worth-Arlington, TX | 12.2% | 12.2% | 48.3% | 24.8% |
| Denver-Aurora-Lakewood, CO | 12.7% | 12.7% | 67.9% | 11.7% |
| Detroit-Warren-Dearborn, MI | 2.4% | 2.4% | 69.7% | 26.2% |
| Houston-The Woodlands-Sugar Land, TX | 16.2% | 16.2% | 37.8% | 26.5% |
| Indianapolis-Carmel-Anderson, IN | 2.2% | 2.2% | 75.4% | 18.6% |
| Kansas City, MO-KS | 4.1% | 4.1% | 75.5% | 16.4% |
| Las Vegas-Henderson-Paradise, NV | 13.8% | 13.8% | 43.7% | 25.3% |
| Los Angeles-Long Beach-Anaheim, CA | 21.3% | 21.3% | 31.5% | 24.9% |
| Miami-Fort Lauderdale-West Palm Beach, FL | 11.7% | 11.7% | 29.6% | 23.2% |
| Minneapolis-St. Paul-Bloomington, MN-WI | 2.2% | 2.2% | 79.1% | 15.8% |
| New York-Newark-Jersey City, NY-NJ-PA | 8.9% | 8.9% | 48.4% | 28.3% |
| Philadelphia-Camden-Wilmington, PA-NJ-DE-MD | 3.8% | 3.8% | 66.1% | 25.7% |
| Phoenix-Mesa-Scottsdale, AZ | 17.8% | 17.8% | 57.1% | 13.5% |
| Portland-Vancouver-Hillsboro, OR-WA | 5.4% | 5.4% | 74.7% | 13.9% |
| San Antonio-New Braunfels, TX | 42.7% | 42.7% | 33.8% | 11.3% |
| San Diego-Carlsbad, CA | 17.5% | 17.5% | 47.5% | 20.5% |
| San Francisco-Oakland-Hayward, CA | 9.9% | 9.9% | 41.9% | 37.5% |
| San Jose-Sunnyvale-Santa Clara, CA | 12.7% | 12.7% | 32.3% | 43.5% |
| Seattle-Tacoma-Bellevue, WA | 4.4% | 4.4% | 65.3% | 25.4% |
| Washington-Arlington-Alexandria, DC-VA-MD-WV | 4.2% | 4.2% | 46.5% | 37.9% |

Footnote**:** Proportion of total of Non-Hispanic White and Hispanic (US and foreign-Born) populations

**Appendix Table 2: Proportion of Workers Employed in Essential Industries by Hispanic/Latino Ethnicity and Nativity. U.S. non-institutionalized employed population living in 27 large MSAs* (2015-2019 American Community Survey)**

|  | **US-Born Hispanic**  **median [IQR]** | **Foreign-Born Hispanic**  **median [IQR]** | **Non-Hispanic White**  **median [IQR]** | **Total**  **median [IQR]** |
| --- | --- | --- | --- | --- |
| **Essential Workers (Industry)** | 81.1%  [78.6% to 82.1%] | 86.9%  [83.1%, 87.7%] | 78.2%  [74.5%, 80.2%] | 81.2%  [78.3%, 83.5%] |

Footnote**:** numbers are median [Interquartile range: Quartile 1, Quartile 3]. Categorization of essential and high risk are not mutually exclusive *part of BCHC

**Appendix Figure 2: Proportion of Workers in Essential Industries by MSA, Ethnicity and Nativity**
